# Genome-Wide Identification and Expression Analysis of ESPs and NSPs Involved in Glucosinolate Hydrolysis and Insect Attack Defense in Chinese Cabbage (*Brassica rapa* subsp. *pekinensis*)

**DOI:** 10.3390/plants12051123

**Published:** 2023-03-02

**Authors:** Danni Han, Jingru Tan, Zhichen Yue, Peng Tao, Juanli Lei, Yunxiang Zang, Qizan Hu, Huasen Wang, Shizhong Zhang, Biyuan Li, Yanting Zhao

**Affiliations:** 1 Institute of Vegetables, Zhejiang Academy of Agricultural Sciences, Hangzhou 310021, China; 2 State Key Laboratory of Crop Biology, College of Life Science, Shandong Agricultural University, Taian 271018, China; 3 Key Laboratory for Quality Improvement of Agricultural Products of Zhejiang Province, College of Agricultural and Food Science, Zhejiang A&F University, Hangzhou 311300, China; 4 Engineering Laboratory of Genetic Improvement of Horticultural Crops of Shandong Province, College of Horticulture, Qingdao Agricultural University, Qingdao 266109, China

**Keywords:** *Brassica rapa*, *ESP*, *NSP*, glucosinolate hydrolysis, insect attack response

## Abstract

Glucosinolates are secondary plant metabolites that are part of the plant’s defense system against pathogens and pests and are activated via enzymatic degradation by thioglucoside glucohydrolases (myrosinases). Epithiospecifier proteins (ESPs) and nitrile-specifier proteins (NSPs) divert the myrosinase-catalyzed hydrolysis of a given glucosinolate to form epithionitrile and nitrile rather than isothiocyanate. However, the associated gene families have not been explored in Chinese cabbage. We identified three *ESP* and fifteen *NSP* genes randomly distributed on six chromosomes in Chinese cabbage. Based on a phylogenetic tree, the *ESP* and *NSP* gene family members were divided into four clades and had similar gene structure and motif composition of *Brassica rapa* epithiospecifier proteins (*BrESPs*) and *B. rapa* nitrile-specifier proteins (*BrNSPs*) in the same clade. We identified seven tandem duplicated events and eight pairs of segmentally duplicated genes. Synteny analysis showed that Chinese cabbage and *Arabidopsis thaliana* are closely related. We detected the proportion of various glucosinolate hydrolysates in Chinese cabbage and verified the function of BrESPs and BrNSPs in glucosinolate hydrolysis. Furthermore, we used quantitative RT-PCR to analyze the expression of *BrESPs* and *BrNSPs* and demonstrated that these genes responded to insect attack. Our findings provide novel insights into BrESPs and BrNSPs that can help further promote the regulation of glucosinolate hydrolysates by ESP and NSP to resist insect attack in Chinese cabbage.

## 1. Introduction

Chinese cabbage (*Brassica rapa* subsp. *pekinensis*) is a leafy cruciferous vegetable in the Brassicaceae family that originated in China, where it has a long history of cultivation. Owing to its rich germplasm resources, high yield per unit area, and easy cultivation, Chinese cabbage is regarded as one of the most important *Brassica* vegetable crops grown in Asia [1]. Glucosinolate constitutes a large group of non-volatile nitrogen- and sulfur-containing secondary metabolites mainly found in Brassicaceae [2,3] that share a chemical structure consisting of a β-d-glucopyranose residue linked via a sulfur atom to a (Z)-N-hydroximinosulfate ester and a variable R group derived from various amino acids. Glucosinolates with R groups derived from Ala, Leu, Ile, Met, or Val are classified as aliphatic glucosinolates; those derived from Phe or Tyr are classified as aromatic glucosinolates; and those derived from Trp are classified as indole glucosinolates. Additionally, most of the R groups are elongated by one or more methylene moieties with a wide variety of transformations, including hydroxylation, O-methylation, desaturation, glycosylation, and acylation that are responsible for the chemical diversity of glucosinolates [4]. Glucosinolate is an important part of the defense system in cruciferous plants and is related to myrosinase, a member of the glucosinolate hydrolase family. When tissue damage, such as mechanical damage, infection, or pest attack occurs, myrosinase released from separate storage compartments [5] cleaves the thioglucosidic linkage of glucosinolate to yield an unstable aglycone intermediate (thiohydroximate-O-sulfonate) [6] that spontaneously produces toxic breakdown products, such as isothiocyanate (ITC) and nitriles. These products have been described as a ‘mustard oil bomb’ and provide protection against bacteria, fungi, insects, and other herbivores [7]. ITC can induce toxicity, growth inhibition, or feeding deterrence to a wide range of potential plant pests. Interestingly, certain specialist insects can exploit ITC as chemical cues to identify their sole host plants, while nitriles might function as signals for lepidopteran larvae parasitoids.

In the presence of specifier proteins, the myrosinase-catalyzed production of thiohydroximate-O-sulfonate can be diverted to form nitriles (NITs), epithionitriles (ENTs), or thiocyanates, depending on the type of specifier protein and chemical structure of the aglucone side chain. Specifier proteins do not act on glucosinolate but are thought to convert glucosinolate aglucones released by myrosinases into non-ITC products [8]. Epithiospecifier proteins (ESPs) are a well-studied type of specifier protein first isolated from *Crambe abyssinica* in 1973 and originally described as 35–40 kDa proteins that promote the formation of ENTs from alkenyl glucosinolates upon myrosinase-catalyzed glucosinolate hydrolysis, rather than ITC [9,10,11]. In addition, ESP promotes the formation of simple NITs from other glucosinolates. Lambrix et al. [12] identified and cloned one *ESP* gene from the Landsberg erecta ecotype of *A. thaliana* by quantitative trait locus mapping and reported that heterologously expressed AtESP could convert glucosinolates into ENT and NIT in the presence of myrosinase. Overexpression of *B. oleracea ESP* in *A. thaliana* also changed the composition of glucosinolate metabolites, resulting in an increase in 4-methoxy-indole-3-acetonitrile and a significant decrease in 1-isothiocyanato-4-methanesulfinyl-butane [13].

ESP is not the only protein responsible for NIT formation. Nitrile-specifier proteins (NSPs) were first identified in *Pieris rapae* and can redirect glucosinolate hydrolysis toward NIT formation, circumventing ITC generation. Based on the assumption that ESP-independent nitrile formation is due to a protein that is similar to ESP, Burow et al. [14] conducted a BLASTN search with the *A. thaliana Ler ESP* cDNA as a query and found that the Kelch proteins that are encoded by six *A. thaliana* genes had 50–60% amino acid sequence identity with ESP. After using NSP activity assay as a screen, the remaining five candidate genes were named *AtNSP1/2/3/4/5* according to the homology with the *AtESP* sequence. AtNSP1 was found to be responsible for constitutive and herbivore-induced NIT formation in rosette leaves. Further research has demonstrated that different AtNSPs are responsible for NIT formation in various organs. AtNSP2 is responsible for NIT formation in seeds; AtNSP1 is responsible for NIT formation in seedlings; and NSP1 and NSP3 are responsible for NIT formation in roots [15]. Sequence and protein structure analysis indicated that ESP shares high sequence similarity with NSP, and both contain four or five Kelch domains (PF01344), whereas some NSP proteins contain one or two lectin-associated jacalin domains at the N-terminus [14]. Both ESP and NSP divert the unstable intermediate to nitriles rather than more effective ITC, which raises the question of why plants produce a large set of specifier proteins but less toxic products. Although NITs are reported to serve as volatile signals related to plant–herbivore interactions, further exhaustive functionality studies need to be conducted.

In this study, three novel *ESP* and fifteen *NSP* family members were identified in Chinese cabbage. The number of genes identified is approximately three times greater than that identified in *A. thaliana*. The validated *BrESP* and *BrNSP* genes were characterized using their chromosomal location, phylogeny, collinearity, genetic structure, conserved protein domain, and cis-acting elements. Additionally, we analyzed the activity of *BrESP* and *BrNSP* in response to glucosinolate hydrolysates in Chinese cabbage and the expression levels that were induced by herbivores. Our findings serve as a genetic and biochemical basis for future studies to characterize the function of nitriles as well as specifier protein in plants.

## 2. Results

### 2.1. Identification of ESP and NSP Gene Family Members

**Figure 1:**
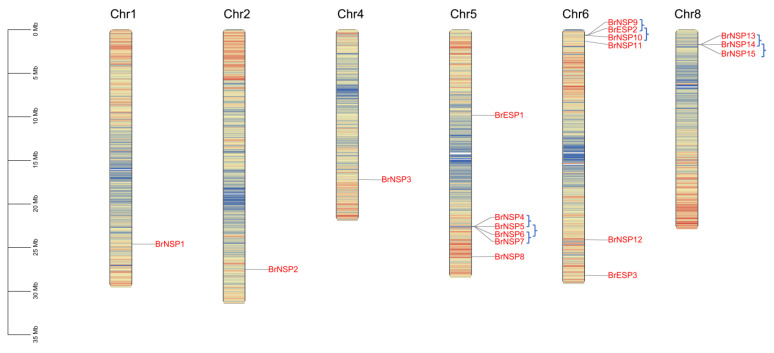
Chromosomal location of *ESP* and *NSP* genes in the Chinese cabbage genome. Tandemly duplicated genes are represented by blue brackets.

Three putative *BrESP* and fifteen putative *BrNSP* genes were identified in the *B. rapa* genome (Brara_Chiifu_V3.5) using HMMER and BLASTP searches. The distinction between *BrESPs* and *BrNSPs* is based on their distance from *AtESP* and *AtNSPs* in the phylogenetic tree. These candidate genes were named based on their localization in the *B. rapa* genome, which was clarified using chromosomal localization analysis and widely accepted nomenclature. *BrESPs* and *BrNSPs* were unevenly distributed on chromosomes 1, 2, 4, 5, 6, and 8. Among them, one *BrESP* and five *BrNSPs* were found on chromosome 5; two *BrESPs* and four *BrNSPs* were found on chromosome 6; three *BrNSPs* were found on chromosome 8; and one *BrNSP* was found on chromosomes 1, 2, and 4. Moreover, we identified seven tandem duplication events containing nine *BrESP* genes and one *BrNSP* gene on chromosomes 5, 6, and 8 (Figure 1). The predicted BrESP and BrNSP proteins are composed of 224–1058 amino acids, with calculated molecular weights ranging from 24.66 kDa to 115.57 kDa (Table 1). Most candidate proteins were acidic (isoelectric point < 7), with only BrNSP9 predicted to be alkaline (isoelectric point > 7). The instability index showed that all BrESP and BrNSP proteins, except for BrNSP1, were predicted to be unstable (instability index > 40). The grand average of hydropathy (GRAVY) value of all candidate proteins was <0, suggesting that these proteins are hydrophilic. In addition, most of the candidate proteins were predicted to be located in the nucleus, except for *BrNSP13* and *BrNSP14*, which were predicted to be located in the cell wall.

### 2.2. Evolutionary Relationships and Collinearity among ESP and NSP Genes

**Figure 2:**
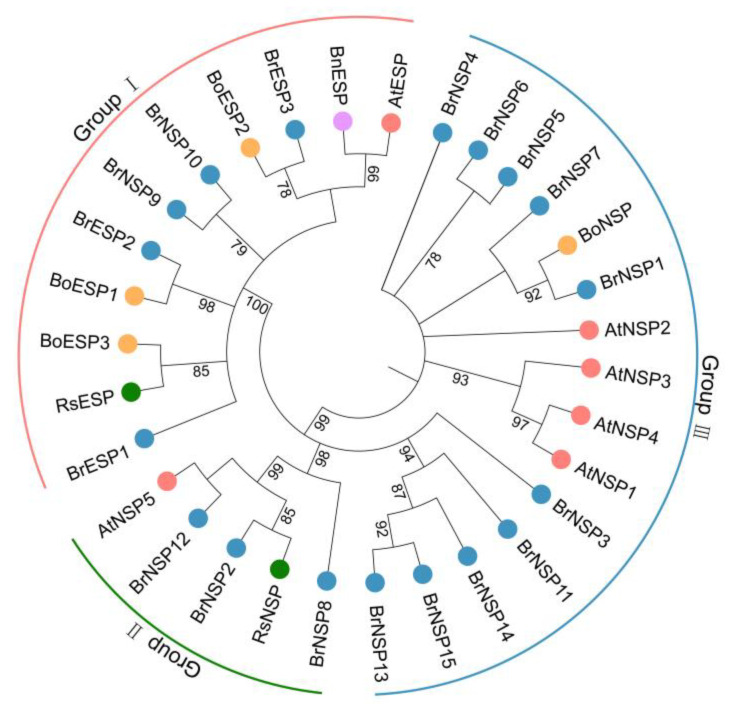
Phylogenetic tree of ESP and NSP proteins from *A. thaliana*, Chinese cabbage, *B. oleracea*, *B. napus*, and *R. sativus*. The phylogenetic tree was plotted using MEGA7 and the maximum likelihood method with 1000 bootstrap replicates.

To analyze the evolutionary relationships among *ESP* and *NSP* genes, we performed phylogenetic analysis of the conserved sequences of 31 ESP and NSP proteins from *A. thaliana*, *Brassica rapa*, *Brassica oleracea*, *Brassica napus*, and *Raphanus sativus*. The phylogenetic tree divided ESP and NSP proteins into three categories according to the evolutionary distance between genes (Figure 2). Group I included all the ESP proteins: one ESP from *A. thaliana*, one from *B. napus*, three from Chinese cabbage, and three from *B. oleracea*. Interestingly, there were two BrNSPs (BrNSP9 and BrNSP10) in this group. Group II included one NSP from *A. thaliana* (AtNSP5), one from *R. sativus*, and three from Chinese cabbage. Group III included four NSP members from *A. thaliana* (*AtNSP1-4*), one from *R. sativus*, thirteen from Chinese cabbage, and one from *B. oleracea* (Figure 2).

**Figure 3:**
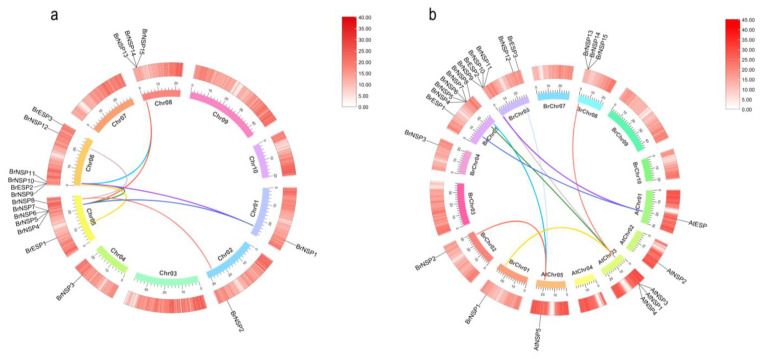
Schematic of the synteny relationships of *ESP* and *NSP* genes. (**a**) Evolutionary analysis of *ESP* and *NSP* genes in Chinese cabbage; (**b**) collinearity analysis of *ESP* and *NSP* genes between *Arabidopsis thaliana* and Chinese cabbage. The outermost circle shows the density of each chromosome, and the inner circle shows the chromosome number. The colored lines represent collinear *ESP* and *NSP* gene pairs.

Gene duplication is an important factor in functional gene differentiation and amplification [16]. We detected eight pairs of segmental duplication events: *BrESP1* and *BrNSP9*, *BrNSP1* and *BrNSP4*, *BrNSP1* and *BrNSP11*, *BrNSP2* and *BrNSP8*, *BrNSP4* and *BrNSP11*, *BrNSP8* and *BrNSP12*, *BrNSP4* and *BrNSP13*, and *BrNSP11* and *BrNSP13* (Figure 3a). Some *BrNSP* genes, particularly *BrNSP4*, *BrNSP8*, *BrNSP11*, and *BrNSP*, were related to at least two pairs of homologs, indicating that gene duplication events played an important role in the differentiation of *BrESP* and *BrNSP* in the Chinese cabbage genome.

The Ka/Ks ratio of a homologous gene pair is an indicator of selection pressure during gene evolution, with Ka/Ks ratios < 1.0 indicating a lower selection pressure [17]. The eight homologous gene pairs displayed Ka/Ks ratios < 1.0 (Table 2), indicating that these genes were subjected to purification selection pressure (Figure 3, Table 2).

To further identify the homologs of the three *BrESP* and fifteen *BrNSP* genes between Chinese cabbage and other plant species, the synteny of *A. thaliana* with Chinese cabbage was analyzed using MCScanX. Synteny analysis of *ESP* and *NSP* genes in these two species showed strong collinearity, despite the occurrence of chromosomal rearrangements or gene duplication (Figure 3b).

### 2.3. Conserved Motifs and Gene Structures of BrESP and BrNSP Genes

**Figure 4:**
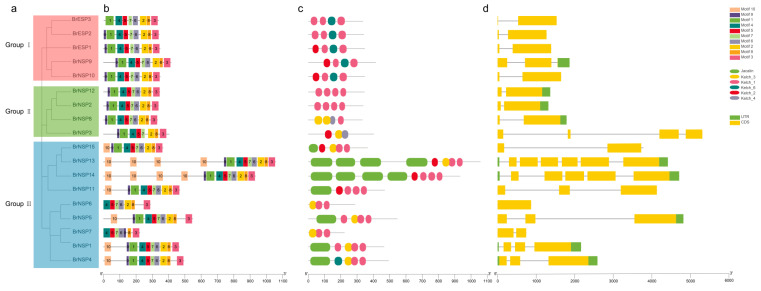
Gene structure of *ESP* and *NSP* gene family members in Chinese cabbage (**a**) Phylogenetic tree of the *ESP* and *NSP* gene families. Branches of the same color represent the same subgroup. (**b**) Motifs 1–10 are represented by different colored boxes. (**c**) Distribution of protein domains. (**d**) Distribution of introns and exons in *ESP* and *NSP* gene families. The green and yellow boxes and gray lines represent the UTRs, exons, and introns, respectively. The length of each nucleotide or protein sequence can be estimated using the scale at the bottom.

To illustrate the diversity of ESP and NSP proteins in Chinese cabbage, the motifs and domains were predicted based on their phylogenetic relationships. Overall, the protein structure of these 18 members was relatively conserved, with similar motifs and 3–4 Kelch domains distributed at the C-termini (Figure 4b,c). The members of Groups I and II contained motifs 1–9 (BrESP3 did not have motif 9, BrNSP3 did not have motif 6), and the overall order was the same, indicating that members within the two subgroups may have similar functions. Group III proteins had motif 10, which was different from those in Groups I and II. BrNSP1, BrNSP4, BrNSP5, BrNSP11, BrNSP13, BrNSP14, and BrNSP15 in Group III had motif 10 at their N-termini, representing a jacalin domain. Among them, BrNSP13 and BrNSP14 had four copies of motif 10 (jacalin domains), which may indicate that their function is stronger than that of other members. They all had a similar Kelch domain arrangement at their C-termini. To better understand the structural diversity among the ESP and NSP genes, exon–intron structures were analyzed (Figure 4d). The number of exons in *ESPs* and *NSPs* ranged from two to six. Genes in Groups I, II, and III contained two exons, 2–4 exons, and 1–6 exons, respectively. Members with similar protein domains and exon numbers were broadly consistent, demonstrating the reliability of the data.

### 2.4. Cis-Element Analysis of BrESP and BrNSP Promoters

**Figure 5:**
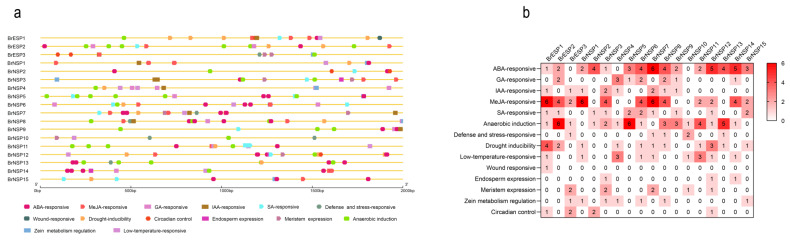
Cis-acting elements in *BrESP* and *BrNSP* genes (**a**) Distribution of cis-acting elements in the promoter region, defined as −2000 bp from the initiation codon. The different colored blocks represent the different types of cis-acting elements and their locations in each *BrESP* and *BrNSP* gene. (**b**) Statistics of the 14 cis-acting elements in the *BrESP* and *BrNSP* genes. The different colors and numbers in the grid indicate the numbers of different cis-acting elements in the *BrESP* and *BrNSP* genes.

Cis-elements upstream of *ESPs* and *NSPs* play important roles in the gene functions involved in plant development and stress response. To better understand the gene function and transcriptional regulatory mechanisms of *BrESPs* and *BrNSPs*, cis-acting elements 2000 bp upstream of the translation initiation site were predicted for all *BrESP* and *BrNSP* genes using the PlantCARE database [18]. As shown in Figure 5, fourteen representative cis-acting elements were selected, including five hormone response elements (abscisic acid, auxin, methyl jasmonate, ethylene, and salicylic acid), inducibility, low-temperature response, wound response, and anaerobic induction, as well as four plant development elements (endosperm expression, meristem expression, circadian control, and zein metabolism regulation). Among these, the cis-acting elements for methyl jasmonate (MeJA) and abscisic acid (ABA) responsiveness and anaerobic induction were enriched in the promoters of most *BrESP* and *BrNSP* genes.

### 2.5. In Vivo Glucosinolate Hydrolysis Assays in Chinese Cabbage Pure Lines

**Figure 6:**
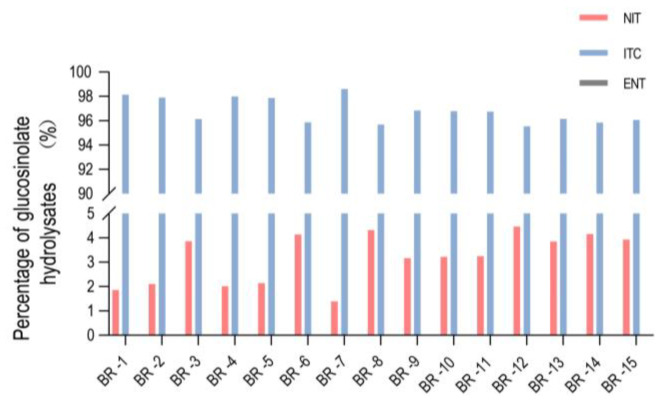
Proportion of glucosinolate metabolites in Chinese cabbage.

To illustrate the activities of BrESPs and BrNSPs in Chinese cabbage, protein extracts of 15 pure Chinese cabbage lines were incubated with the exogenous glucosinolate, sinigrin, and the breakdown products of sinigrin being measured using gas chromatography. The results showed that the ITC content in these 15 cabbage varieties was higher than that of NIT. The content of NIT in all 15 Chinese cabbage materials was only between 0.5 and 2 mM, while that of ITC was higher than 30 mM, with a maximum of 48.887 mM (Table S1). Interestingly, ENT was not detected in our assay. Among the lines analyzed, BR-6, BR-8, BR-12, and BR-14 had slightly higher NIT levels than the other lines (Figure 6a), indicating variation in the expression of *BrESP* and *BrNSP* genes in Chinese cabbage lines.

### 2.6. Expression of BrESP and BrNSP in Response to Spodoptera Littoralis Attack

**Figure 7:**
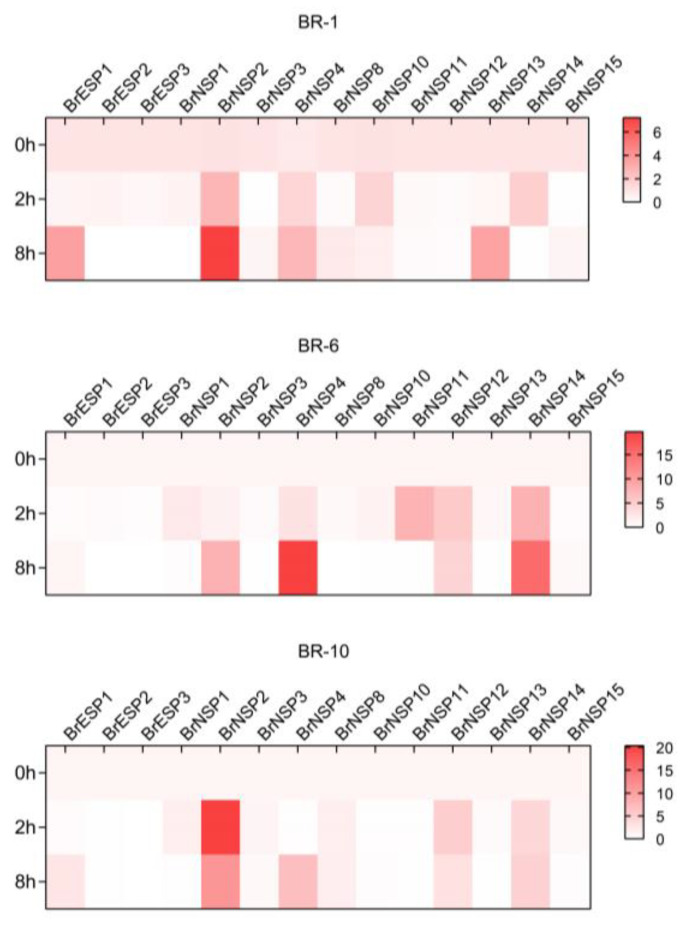
Expression of *BrESPs* and *BrNSPs* in three varieties of Chinese cabbage after insect attack.

To clarify the response of *BrESPs* and *BrNSPs* to insect attacks in Chinese cabbage, gene expression was measured after Chinese cabbage was predated on by *Spodoptera littoralis*. The results showed that the expression of *BrESP1*, *BrNSP2*, *BrNSP4*, *BrNSP12*, *BrNSP13*, and *BrNSP14* was highly induced but varied among the different Chinese cabbage pure lines. By contrast, the expression of other *BrESPs* and *BrNSPs* did not significantly change. In BR-1, the expression of *BrESP1*, *BrNSP4*, and *BrNSP13* noticeably increased; in BR-6, *BrNSP2*, *BrNSP4*, *BrNSP11*, and *BrNSP14* were highly expressed; and in BR-10, *BrNSP2*, *BrNSP4*, *BrNSP12*, and *BrNSP14* were all highly expressed (Figure 7). These results suggest that *BrNSP* genes respond more strongly than *BrESP* genes to insect attack and that different *BrNSPs* are induced in Chinese cabbage pure lines.

## 3. Discussion

### 3.1. Identification and Characterization of ESP and NSP Genes

Although most studies have focused on highly reactive and biologically active ITCs, there is growing evidence that suggests that NITs are involved in a complex network of interactions between plants and their biotic environment. Plant and insect specifier proteins, such as ESP and NSP, promote the rearrangement of aglucone to NITs and ENTs.

In 1973, Tookey [10] separated the protein factor responsible for ENT formation in the seeds of *Crambe abyssinica* from myrosinase and named it ESP. Subsequently, ESP was identified in other species of Brassicaceae [12,19,20,21,22]. ESP was purified from *Brassica napus* [20,21], and its partial amino acid sequence was analyzed [20]. Through quantitative trait locus (QTL) mapping, *ESP* was cloned from the Landsberg erecta ecotype of *A. thaliana*, and its amino acid sequence was 80% identical to the partial sequence of *B. napus* ESP [12]. An ESP with an amino acid sequence 77% identical to that of *A. thaliana* ESP was identified in *B. oleracea* [22], and published genomic data [23] helped identify three ESPs via sequence alignment with the AtESP amino acid sequence in *B. oleracea* [19]. Phylogenetic analysis, *A. thaliana* mutant screening, recombinant protein characterization, and expression QTL mapping identified five NSPs with 50–60% identity to the amino acid sequence of AtESP [14]. In addition, only one NSP protein was identified in *B. oleracea* by multiple alignment [24]. In our assay, we predicted three *BrESP* and fifteen *BrNSP* genes (Table 1) using BLASTP alignment with the Hidden Markov Model of the Kelch domain and sequences of *AtESPs* and *AtNSPs* from Chinese cabbage genomic data, which is approximately triple the number of *ESP* and *NSP* genes present in *A. thaliana.* This difference may be due to three genome-wide duplication events that occurred during Chinese cabbage evolution [25].

The three *BrESP* genes and fifteen *BrNSP* genes were divided into three groups based on the evolutionary distance between genes (Figure 2) because genes in the same clade might have similar or complementary physiological functions. Group I contained all the ESPs, indicating that the members in this group could promote the formation of ENTs. We predicted that the physiological functions of BrNSP9 and BrNSP10, but these predictions need to be further confirmed by subsequent biochemical experiments. Based on the results of evolutionary tree and protein structure analysis, we hypothesized that the function of NSPs in Group II was similar to that of AtNSP5, which is responsible for NIT formation rather than ENT but lacks N-terminus jacalin domains compared with other AtNSPs. Protein structure analysis showed that BrESPs and BrNSPs contained three to four Kelch domains (Figure 4c), which is consistent with the results obtained from *A. thaliana.* The Kelch repeat domain forms a β-propeller structure and functions as a protein–protein interacting domain that binds substrates for ubiquitin-mediated protein degradation [26], which was also confirmed by the analysis of the predicted structure of the *A. thaliana* ESP [27,28]. These results support the hypothesis that ESP is an allosteric cofactor for myrosinase. The proteins Kelch repeat and LOV Kelch protein 2 were identified as blue light photoreceptors important for the regulation of the circadian clock and photoperiodic flowering [29,30,31]. Similarly, glucosinolate and its metabolism participate in the circadian clock and photoperiodic flowering, indicating that *BrESPs* and *BrNSPs* might be involved in pathways other than glucosinolate hydrolysis [32,33,34].

In addition, four AtNSP and seven BrNSP proteins were found to contain lectin-like jacalin domains (Figure 4c). Burow et al. [14] found that the jacalin domain at the N-terminus of AtNSP has high amino acid sequence similarity to putative *A. thaliana* myrosinase-binding proteins (MBPs), as well as the same ancestor. Despite these structural differences, all five AtNSPs exhibited NIT-forming activity but no ENT- or thiocyanate-forming activity. MBPs have long been associated with the glucosinolate-myrosinase system and can form stable complexes with myrosinase in *Brassica* species [35,36,37]. However, the biological functions of these compounds remain unknown. The antisense suppression of MBP in transgenic *B. napus* prevented myrosinase complex formation but did not affect the hydrolysis product profile, indicating that the myrosinase complex has an extra function compared to that of the glucosinolate hydrolytic enzyme. Based on the similarity of the jacalin domains to MBPs and the absence of Kelch domains in MBPs, a putative function of the jacalin domains might affect the interaction between NSPs and myrosinase [14]. Given that ESP and AtNSP5 can act as specifier proteins during glucosinolate hydrolysis despite lacking a jacalin domain, this domain may affect other NSP characteristics, such as tissue localization and regulation. Overall, whether and how the Kelch and jacalin domains contribute to the catalytic activity of the NSPs or to the function of these proteins in plants requires further research [14].

### 3.2. Evolutionary Relationship of Kelch and Jacalin Domains

Phylogenetic analysis of the Kelch domains of *A. thaliana* ESP and NSPs showed that the evolutionary relationship between AtNSP1, AtNSP2, AtNSP3, and AtNSP4 was very close [14]. In contrast, *AtNSP5* appears to be a single-copy gene in *A. thaliana* that has homologues in all fungal, algal, bryophyte, and higher plant species tested. This suggests that *AtNSP5* may encode the ancestral function from which the glucosinolate-related activity is derived. Our phylogenetic tree also showed that AtNSP5, BrNSP2/8/12, and RsNSP are independently located in a separate evolutionary branch (Figure 2). Interestingly, AtNSP1/2/3/4 contain jacalin domains that are not present in AtNSP5 or AtESP or in the ancestral protein encoded by *AtNSP5* [14]. Phylogenetic analysis with the jacalin domains showed that these domains were derived from putative MBPs. In the protein domain analysis, we found that most BrNSPs in Group III have a jacalin domain, which is related to the fact that they and AtNSP1/2/3/4 are located in the same evolutionary branch (Figure 4c). The jacalin domain in BrNSPs may be the same as AtNSP5.

### 3.3. Intra- and Inter-Specific Relationships among BrESP and BrNSP Genes

Gene duplication events are important for plant evolution and gene family expansion [38]. Studies have shown that 70–80% of angiosperms have experienced gene duplication or polyploid events [39]. From a biological evolutionary perspective, fragment replication, tandem duplication, and translocation are effective at generating new genes and developing resistance against foreign invaders [40]. Seven pairs of tandem duplicated genes and eight pairs of segmental duplication genes were found among the three *BrESP* and fifteen *BrNSP* genes in Chinese cabbage (Figure 3a), indicating that gene duplication events have greatly promoted the expansion of the *BrESP* and *BrNSP* gene families. In addition, there was a collinear relationship between Chinese cabbage and *A. thaliana*, which identified two pairs of *ESP* and six pairs of *NSP* genes (Figure 3b). Orthologous genes encode proteins with common biological functions. These results suggest that *ESP* and *NSP* genes in *A. thaliana* function in regulating glucosinolate metabolites, which may have been inherited from Chinese cabbage during evolution.

### 3.4. ESPs and NSPs Regulate GLS Hydrolysis

Witzel et al. [19] detected the hydrolysis products of GLS in four *B. oleracea* genotypes. They reported that NITs were formed mainly in shoots of kohlrabi and broccoli; however, no or low levels of ETNs were found. The ETNs were most abundant in white cabbage shoots and were associated with 74% of the degradation products, while roots formed more NITs (70%) than ETNs (20%). Shoots of red cabbage released mainly ITCs (51%), followed by ETNs (41%), while root tissue released mainly NITs (59%) and low amounts of ETNs (5%). A large amount of ITCs and a small amount of NIT were detected in the fifteen Chinese cabbage varieties tested, while ENT was not detected. This was different from the proportion of GLS hydrolysis products detected in four *B. oleracea* genotypes; therefore, we speculate that this may be related to the physiological characteristics of Chinese cabbage and *B. oleracea*. However, when measuring the effect of AtNSP1 on the hydrolysis of GLS in vitro, it was reported that when purified NSP1 or myrosinase was added to the system, a large amount of ITC and a very small amount of simple nitrile and ENT were detected [14], suggesting that BrNSP may have the same product proportion when hydrolyzing GLS. In addition, NSP1 and NSP2 in germinating seeds and developing seedlings can regulate glucosinolate levels in and make contributions to glucosinolate turnover during the seed–seedling transition [41]. We believe that BrNSP1 and BrNSP4, which are closely related to AtNSP1 and AtNSP2, may play a similar role in glucosinolate turnover. However, there are few studies on the functions of ESP and NSP in insect defense, and we will continue to study them in the future.

### 3.5. BrESP and BrNSP Genes Are Involved in Insect Defense Mechanisms

Simple nitriles serve as volatile signals in direct and indirect defense responses against insects; the proportion of nitriles formed from the endogenous glucosinolates of the feeding *Col-0* leaves increased dramatically, accompanied with an induction of the expression of specifier protein. In the present assay, the expression levels of *BrESP* genes and *BrNSP* genes were induced upon *S. littoralis* attack. However, it seems that *BrNSP* genes were mainly induced when compared with *BrESP* genes, and the members of *BrNSPs* showed various expression patterns in different Chinese cabbage lines.

Burow et al. [14] found that the proportion of accumulated NITs in the leaf homogenate of the Landsberg erecta ecotype of *A. thaliana* fed to *P. rapae* larvae was twice that of untreated leaves; the addition of exogenous allyl glucosinolate did not form ENT, and ESP did not exhibit activity, which was similar to our results (Figure 6 and Figure 7). Therefore, it was hypothesized that NSP with an ESP-like structure induced the synthesis of NIT. In the three randomly selected Chinese cabbage lines, we detected NIT but no ENT in the glucosinolate metabolites, and the expression of *BrNSP2* and *BrNSP4* was significantly higher than that of *BrESP1*. Therefore, the activity of *NSP* could have been much higher than that of *ESP* before Chinese cabbage was attacked by insects. In addition, *AtESP* was not induced in the leaves 24 h after insect attack, whereas *AtNSP1* expression was upregulated. The QRT results of this study showed that *BrESP* was barely induced after Chinese cabbage was attacked by *S. littoralis*, but the expression of *BrESP1*, *BrNSP2*, *BrNSP4*, *BrNSP12*, *BrNSP13*, and *BrNSP14* was significantly upregulated, which was similar to that observed for *A. thaliana*. Furthermore, when the roots, seeds, and seedlings of *A. thaliana* were damaged, most of the NIT formation activity was regulated by NSP [15]. This further verifies that the increase in NITs after plant leaves are attacked by herbivores is mainly determined by *NSPs* and has only a weak relationship with *ESPs*.

Analysis of the promoter region revealed that most of the cis-element components in *BrESP* and *BrNSP* genes were associated with phytohormones, especially MeJA and ABA, which actively regulate plant defense and glucosinolate metabolism (Figure 5a) [42,43]. This result demonstrated that the application of exogenous MeJA can increase glucosinolate accumulation and systemic defense against insects. High indole glucosinolate (IGS) content in *A. thaliana aba1-1* mutants increases defense responses to *Myzus persicae* [44]. Given that ESP- and NSP-dependent glucosinolate hydrolysis play an important role in the interaction between Chinese cabbage and insects, we hypothesized that glucosinolate hydrolysis is directly regulated by MeJA and ABA and acts downstream of phytohormone signaling pathways, which are activated by herbivore attacks and involved in Chinese cabbage defense against herbivory.

## 4. Materials and Methods

### 4.1. Plant Materials and Sample Collections

Fifteen Chinese cabbage cultivars BR-1 to BR-15 were analyzed in the present study. They are important Chinese cabbage germplasm resources collected by Zhejiang Academy of Agricultural Science (ZAAS, Hangzhou, China) (Table S2). Seeds of the fifteen cultivars were sown into a 50-holed plastic tray, set at 25 °C with a natural photoperiod. Ten days-old plants were used for the experiments. Three pure lines were randomly selected, each of which selected Chinese cabbage in uniform condition, and six fall armyworms purchased from the market will be placed evenly on the leaves. The surrounding parts of the bitten leaves were collected at 0 h, 2 h, and 8 h after being bitten by insects. All the collected samples were immediately frozen in liquid nitrogen and stored at −80 °C.

### 4.2. Genome-Wide Identification of BrESPs and BrNSPs

Both the *Brassica rapa* genome and protein sequences were retrieved from BRAD (Brassicaceae Database) (http://brassicadb.cn/#/, accessed on 10 February 2022). To identify Chinese cabbage genes, BLASTP was performed using *A. thaliana* ESP and NSP protein sequences retrieved from The *A. thaliana* Information Resource (TAIR, https://www.arabidopsis.org/, accessed on 10 February 2022), and the Hidden Markov Model (HMM) profile of the respiratory burst Kelch domain (PF01344) downloaded from the pfam (http://pfam.xfam.org/, accessed on 10 February 2022) as queries. All the putative BrESPs and BrNSPs were further confirmed through the Pfam database and the SMART database for conserved domains. The chromosomal location map of *BrESP* and *BrNSP* genes was displayed by the TBtools software [45]. The physicochemical properties of these selected BrESPs and BrNSPs, including their molecular weight, instability index value, isoelectric point (pI) and hydrophilic, were predicted with ExPASy. The Cell-PLoc was subsequently used to predict the subcellular localization of these proteins (http://www.csbio.sjtu.edu.cn/bioinf/Cell-PLoc−2/, accessed on 15 February 2022).

### 4.3. Phylogenetic Analysis and Collinearity Analysis of BrESPs and BrNSPs

The phylogenetic analysis of ESPs and NSPs from *A. thaliana* and Chinese cabbage was performed using ClustalW in MEGA 7.0 (Temple University, Philadelphia, PA, USA) [46] with the maximum likelihood method and the protest analysis being used. The bootstrap was performed with 1000 replicates. Then, the phylogenetic tree was constructed by iTOL (http://itol.embl.de/, accessed on 20 February 2022).

Genomic data for Chinese cabbage and *A. thaliana* were downloaded from the BRAD and Ensembl Plants Database, respectively. Multiple collinear scanning toolkits (MCScanX) with default parameters were used to analyze gene duplication events (http://chibba.pgml.uga.edu/mcscan2/, accessed on 30 February 2022). The syntenic relationship between *ESP* and *NSP* genes in *A. thaliana* and Chinese cabbage genome was determined using the Dual Synteny Plotter tool in TBtools software.

### 4.4. Determination of Gene Structure, and Conserved Motifs

BrESP and BrNSP proteins sequences were submitted to MEME Suite 5.3.3 (https://meme-suite.org/meme/tools/meme, accessed on 9 March 2022) to predict the conserved motifs with default settings for parameters (the maximum number of motifs set to 10). The BrESP and BrNSP gene structure was determined and visualized by TBtools software. The CDD (Conserved Domain Database) of the BrESP and BrNSP was explored by NCBI and visualized by TBtoolos software.

### 4.5. Analysis of Cis-Acting Elements in Gene Promoter

The promoter sequences of the *BrESP* and *BrNSP* genes (2 kb of the 5′ regulatory region upstream of the translation start sites) were obtained by searching the BRAD. PlantCARE (plant cis-acting regulatory element database) (http://bioinformatics.psb.ugent.be/webtools/plantcare/html/, accessed on 14 March 2022) was used to predict the cis-acting elements and visualized in GDSD (http://gsds.gao-lab.org/, accessed on 17 March 2022).

### 4.6. Glucosinolate Assay

The glucosinolates in Chinese cabbage leaves were extracted and measured according to previously described [44] with following modifications. Briefly, nearly 0.1 g of powdered sample (with three biological replicates) was boiled in 10 mL ddH_2_O for 10 min. The supernatant was collected, and the residues were re-extracted with 10 mL boiling ddH_2_O for 10 min. The combined aqueous extract was purified with DEAE-Sephadex A-25 (Sigma-Aldrich, Shanghai, China). The glucosinolates were converted into their desulfo analogus through overnight enzymolysis with aryl sulfatase (Sigma-Aldrich, Shanghai, China); then, the desulfoglucosinolates were eluted with ddH_2_O and analyzed with HPLC. The HPLC system used in present assay was the Waters 600 system equipped with a 2487 UV detector (Waters Corp., Milford, MA, USA). Desulphoglucosinolates were separated on Spherisorb C18 column (Elite Analytical Instruments Co., Ltd., 4.6 mm × 250 mm, Dalian, China) at flow rate of 1.0 mL/min with a gradient elution of mobile phases ddH_2_O (A) and acetonitrile (B) (B:A = 1.5:98.5, *v/v*). Data were given as μ mol/g DW.

### 4.7. RNA Extraction, cDNA Synthesis, and qRT-PCR Analysis

The total RNA of samples was extracted using Plant Total RNA Isolation Kit (Sangon Biotech, Shanghai, China). The first cDNA strand was synthesized by the PrimeScript™ RT reagent Kit with gDNA Eraser (Takara, Dalian, China). The expression of the gene encoding actin-7 (*BraA10g028050.3.5C*) [47] was used as an internal expression control. The gene-specific primers used for the qRT-PCR were listed in Supplementary Table S3. Real-time PCR was performed with the CFX96 real-time PCR machine (BIO-RAD, Berkeley, CA, USA) using TransStart ^®^ Top Green qPCR SuperMix (TransGen, Beijing, China). The 2−ΔΔCT Ct method was used to calculate the relative gene expression level across the samples [48]. Finally, the results were presented as histograms by GraphPad Prism 8 software (GraphPad, San Diego, CA, USA).

## Figures and Tables

**Table 1:** Physiological and biochemical characterization of *ESP* and *NSP* proteins in Chinese cabbage.

Gene Name	Gene ID	Protein Length (aa)	Molecular Weight (KD)	Isoelectric Point	Instability Index	GRAVY	Subcellular Localization
*BrESP1*	*BraA05g016920.3.5C*	348	38.49	5.45	66.35	−0.498	Nucleus
*BrESP2*	*BraA06g000990.3.5C*	343	37.73	5.71	60.23	−0.540	Nucleus
*BrESP3*	*BraA06g044470.3.5C*	337	37.29	6.08	59.32	−0.553	Nucleus
*BrNSP1*	*BraA01g037250.3.5C*	467	51.16	5.40	25.49	−0.316	Nucleus
*BrNSP2*	*BraA02g041120.3.5C*	339	37.12	5.06	76.64	−0.226	Nucleus
*BrNSP3*	*BraA04g024490.3.5C*	403	43.87	5.89	75.88	−0.342	Nucleus
*BrNSP4*	*BraA05g031740.3.5C*	495	54.26	4.64	78.67	−0.268	Nucleus
*BrNSP5*	*BraA05g031760.3.5C*	548	60.46	5.70	76.57	−0.300	Nucleus
*BrNSP6*	*BraA05g031780.3.5C*	289	31.81	5.25	72.77	−0.391	Nucleus
*BrNSP7*	*BraA05g031790.3.5C*	224	24.66	5.04	66.96	−0.373	Nucleus
*BrNSP8*	*BraA05g039060.3.5C*	334	36.14	5.04	64.49	−0.381	Nucleus
*BrNSP9*	*BraA06g000970.3.5C*	415	45.86	8.68	60.39	−0.573	Nucleus
*BrNSP10*	*BraA06g001040.3.5C*	349	38.33	4.91	59.74	−0.494	Nucleus
*BrNSP11*	*BraA06g002280.3.5C*	470	51.57	5.76	69.83	−0.318	Nucleus
*BrNSP12*	*BraA06g037100.3.5C*	346	37.65	5.42	75.69	−0.216	Nucleus
*BrNSP13*	*BraA08g002360.3.5C*	1058	115.57	6.16	72.72	−0.345	Cell wall
*BrNSP14*	*BraA08g002370.3.5C*	934	101.72	6.57	71.47	−0.333	Cell wall
*BrNSP15*	*BraA08g002390.3.5C*	366	40.61	6.09	72.92	−0.329	Nucleus

**Table 2:** Duplications of *ESP* and *NSP* genes in Chinese cabbage.

Gene 1	Gene 2	Ka	Ks	Ka/Ks	Selection Pressure
*BrNSP1*	*BrNSP4*	0.0838655	0.3288921	0.254994	Purifying selection
*BrNSP1*	*BrNSP11*	0.2772172	1.3130947	0.2111174	Purifying selection
*BrNSP2*	*BrNSP8*	0.3699146	1.0279427	0.3598591	Purifying selection
*BrESP1*	*BrNSP9*	0.1815313	0.5013181	0.362108	Purifying selection
*BrNSP4*	*BrNSP11*	0.3016929	1.4593027	0.2067377	Purifying selection
*BrNSP8*	*BrNSP12*	0.3681619	1.1185719	0.3291357	Purifying selection
*BrNSP4*	*BrNSP13*	0.2906193	1.3147322	0.2210483	Purifying selection
*BrNSP11*	*BrNSP13*	0.1786629	0.5385724	0.3317343	Purifying selection

## Data Availability

Not applicable.

## Supplementary Materials

The following supporting information can be downloaded at: https://www.mdpi.com/article/10.3390/plants12051123/s1, Table S1. Experimental data of glucosinolate hydrolysis of fifteen Chinese cabbage varieties. Table S2. Characteristics of fifteen Chinese cabbage varieties. Table S3. Gene-specific primers for qRT-PCR.
